# First Spanish Experience with Stereotactic MR-Guided Adaptive Radiotherapy (SMART) in Borderline Resectable and Locally Advanced Pancreatic Cancer: A Prospective Study

**DOI:** 10.3390/biomedicines13102390

**Published:** 2025-09-29

**Authors:** Daniela Gonsalves, Abrahams Ocanto, Eduardo Meilan, Alberto Gomez, Jesus Dominguez, Lisselott Torres, Castalia Fernández, Macarena Teja, Isabel Garrido, Maria Gonzalez, Miren Gaztañaga, Daniel Herrero, Israel J. Thuissard, Cristina Andreu, Tomas Gonzalez, Jose Antonio González, Jon Andreescu Yagüe, Esther Holgado, Diego Alcaraz, Escarlata López, Maia Dzhugashbli, Luis Glaria, Fernando Lopez-Campos, Esther Dominguez, Jesús Rodriguez Pascual, Eva Maria Lozano Martin, David Sanz-Rosa, Michael D. Chuong, Olivier Riou, Felipe Couñago

**Affiliations:** 1 Department Radiation Oncology, GenesisCare Madrid, Hospital Vithas la Milagrosa, 28010 Madrid, Spain; abrahams.ocanto@genesiscare.es (A.O.); jesus.dominguez@genesiscare.es (J.D.); lisselott.torres@genesiscare.es (L.T.); castalia.fernandez@genesiscare.es (C.F.); macarena.teja@genesiscare.es (M.T.); isabel.garrido@genesiscare.es (I.G.); maria.gonzalez@genesiscare.es (M.G.); felipe.counago@genesiscare.es (F.C.); 2 Department Radiation Oncology, GenesisCare Madrid, Hospital San Francisco de Asis, 28002 Madrid, Spain; miren.gaztanaga@genesiscare.es (M.G.); dzhugashbli.maia@genesiscare.es (M.D.); glaria.luis@genesiscare.es (L.G.); fernando.lopez@genesiscare.es (F.L.-C.); 3 Facultad de Medicina Salud y Deporte, Universidad Europea de Madrid, 28670 Madrid, Spain; israeljohn.thuissard@universidadeuropea.es (I.J.T.); david.sanz@universidadeuropea.es (D.S.-R.); 4 Department Radiation Physicist, GenesisCare Madrid, Hospital Vithas la Milagrosa, 28010 Madrid, Spain; eduardo.meilan@genesiscare.es (E.M.); alberto.gomez@genesiscare.es (A.G.); 5 Department Medical Oncology, HLA Hospital Universitario Moncloa, 28008 Madrid, Spain; dhrivera79@yahoo.es; 6 Facultad de Veterinaria, Universidad Europea de Madrid, 28670 Madrid, Spain; cristina.andreu@univesidadeuropea.es; 7 Department Radiation Oncology, Cancer Center ABC, The American British Cowdray Medical Center, Mexico City 01120, Mexico; drgonzalezonco@gmail.com; 8 Department Radiation Oncology, GenesisCare Seville, 41092 Seville, Spain; jose.gonzalez@genesiscare.es; 9 Department Radiation Oncology, GenesisCare Cordoba, 14012 Cordoba, Spain; 10 Department Medical Oncology, GenesisCare Madrid, Hospital San Francisco de Asis, 28002 Madrid, Spain; esther.holgado@genesiscare.es (E.H.); diego.alcaraz@genesiscare.es (D.A.); 11 Department Radiation Oncology, GenesisCare Málaga, 29018 Málaga, Spain; escarlata.lopez@genesiscare.es; 12 Department Radiology, Hospital Vithas la Milagrosa, 28010 Madrid, Spain; dominguezfm@vithas.es; 13 Vithas Oncoloy Institute, Hospital Vithas la Milagrosa, 28010 Madrid, Spain; rodriguezpje@vithas.es; 14 Department Radiation Oncology, Hospital Universitario Toledo, 45007 Toledo, Spain; emlozano@sescam.jccm.es; 15 Miami Cancer Institute, Baptist Health South Florida, Miami, FL 33176, USA; michaelchu@baptisthealth.netw; 16 Montpellier Cancer Institute (ICM), University Federation of Radiation Oncology of Mediterranean Occitanie, University Montpellier, INSERM U1194 IRCM, 34298 Montpellier, France; olivier.riou@icm.unicancer.fr

**Keywords:** pancreatic cancer, MR-guided radiotherapy, stereotactic body radiotherapy (SBRT)

## Abstract

**Background/Objectives:** In Spain, pancreatic ductal adenocarcinoma (PDAC) is the seventh leading cause of cancer-related death, with only 20% of patients eligible for surgery at diagnosis. For the remaining majority, prognosis is poor and effective non-surgical strategies are needed. Stereotactic MR-guided adaptive radiotherapy (SMART) may facilitate the delivery of ablative doses of radiation safely with low toxicity. This study reports the first national experience in Spain with SMART for patients with borderline resectable (BRPC) or locally advanced pancreatic cancer and evaluates its feasibility, safety, and early clinical outcomes. **Methods:** A prospective observational study was conducted including 28 patients with histologically confirmed BRPC or LAPC treated between August 2023 and December 2024. All patients received induction chemotherapy—mainly FOLFIRINOX (57.1%)—followed by SMART delivered in five fractions (40–50 Gy) using a 0.35T MR-guided linear accelerator. Daily online adaptive recontouring and replanning were performed for all 140 treatment fractions. Toxicities were assessed using CTCAE v5.0, and survival outcomes were estimated using Kaplan–Meier analysis. **Results:** The median patient age was 67 years, and 71.4% of tumors were located in the pancreatic head. At a median follow-up of 7.4 months after SMART (12.25 months from diagnosis), 6-month local progression-free survival (LPFS) was 89.3% from the start of SMART and 82.1% from diagnosis. Distant progression-free survival (DPFS) at 6 and 12 months was 92.9% and 68.2%, respectively. Median progression-free survival (PFS) was 11.5 months, and the median treatment-free interval was 5.7 months. Median overall survival (OS) was not reached; 6- and 12-month OS rates were 89.3% and 74.1%, respectively. Treatment-related toxicity was limited to grade 2 abdominal pain in 14.3% of patients, with no grade ≥3 adverse events attributed to SMART. **Conclusions:** SMART is a feasible and safe treatment modality for BRPC and LAPC in real-world clinical practice. These encouraging early outcomes support further clinical investigation and broader implementation.

## 1. Introduction

Pancreatic ductal adenocarcinoma (PDAC) is one of the most aggressive malignancies worldwide, with a 5-year overall survival (OS) of less than 11% [1]. In Spain, pancreatic cancer is the seventh leading cause of cancer incidence, with projected rates of 21.7 per 100,000 in men and 20 per 100,000 in women by 2025, representing a 5% increase compared with the previous decade [2]. Most patients are diagnosed at an advanced age, commonly over 65 years, and only around 20% are eligible for surgical resection at presentation [3]. These statistics highlight the critical need for improved therapeutic strategies, as the prognosis remains poor despite advances in systemic therapy and surgical techniques.

The management of PDAC is primarily determined by tumor stage and resectability. Tumors are classified as resectable, borderline resectable (BRPC), locally advanced (LAPC), or metastatic, with vascular involvement playing a key role in surgical decision-making [4,5,6,7,8]. Surgery with microscopically negative margins (R0 resection) remains the most important independent prognostic factor, with 5-year survival rates of 26% compared with 8% for patients with margin-positive resections (R1) [4,5]. While venous reconstruction is feasible, arterial resections are associated with higher morbidity and mortality [9]. For patients with BRPC or LAPC, systemic therapy and radiotherapy aim to control local disease and, when possible, convert tumors to resectable status [10,11]. However, despite randomized trials such as CONKO-007 [12] and LAP-07 [13] demonstrating that induction chemotherapy and chemoradiation can increase R0 resection rates, significant improvements in overall survival have not been consistently observed. Conventional intensity-modulated radiotherapy (IMRT) over 5–6 weeks has been the standard approach in LAPC [14], but challenges remain due to tumor radio resistance and proximity to radiosensitive organs such as the duodenum, stomach, and small bowel.

Recent advances in radiotherapy, including stereotactic body radiotherapy (SBRT) and magnetic resonance-guided radiotherapy (MRgRT), have enabled more precise and effective treatment of PDAC. SBRT allows delivery of ablative doses in a limited number of fractions, reducing toxicity compared with conventional chemoradiation [15]. MRgRT further enhances precision through real-time imaging, adaptive planning, and daily target adjustment, which is particularly relevant in anatomically complex regions [16]. Stereotactic MR-guided adaptive radiotherapy (SMART) combines ultrahypofractionated SBRT with daily adaptation to optimize target coverage while sparing surrounding tissues, showing local control rates above 80% with limited grade ≥3 toxicity in early studies [17].

Despite these promising results, no data are currently available on the use of SMART for pancreatic cancer in Spain. Establishing its feasibility, safety, and preliminary efficacy is essential before broader implementation. Therefore, the aim of this prospective observational study was to report the first national experience with SMART in patients with BRPC and LAPC and to assess feasibility, safety, and early outcomes in terms of local progression-free survival (LPFS), distant progression-free survival (DPFS), progression-free survival (PFS), overall survival (OS), and treatment-free interval (TFI).

## 2. Materials and Methods

### 2.1. Study Design and Patient Selection

This prospective, longitudinal, observational (basket-type) study was approved in July 2023 by the Ethics Committee of Hospital Universitario La Princesa (Registry #5289). Between 1 August 2023, and 31 December 2024, 37 patients with histologically confirmed pancreatic ductal adenocarcinoma (PDAC) were initially evaluated, of whom 28 met all eligibility criteria and were included in the study at Vithas La Milagrosa Hospital (Madrid, Spain). Eligible patients had borderline resectable or locally advanced pancreatic cancer (BRPC or LAPC) according to the National Comprehensive Cancer Network (NCCN) guideline (version 2.2024) [8], life expectancy > 6 months, Eastern Cooperative Oncology Group (ECOG) performance status 0–1, completion of at least three months of chemotherapy, and adequate organ function (hemoglobin > 9 g/dL, absolute neutrophil count > 1.5 × 10^9^/L, platelet count > 80 × 10^9^/L, bilirubin < 3× ULN, INR < 1.3 or correctable with vitamin K, AST/ALT < 5× ULN, and serum creatinine < 200 µmol/L). Patients were excluded for MRI-incompatible claustrophobia, direct gastrointestinal invasion, non-adenocarcinoma histology, prior abdominal radiotherapy, or history of metastatic disease. Written informed consent was obtained from all participants.

All included patients underwent pancreatic MRI at least two weeks prior to stereotactic MR-guided adaptive radiotherapy (SMART) and observed a minimum one-week chemotherapy-free interval before and after treatment. Cases were reassessed in a multidisciplinary tumor board, and patients were encouraged to undergo surgical resection if conversion to resectable status occurred after induction SMART; however, no patients proceeded to surgery during follow-up.

### 2.2. Simulation and Treatment Planning

**Figure 1:**
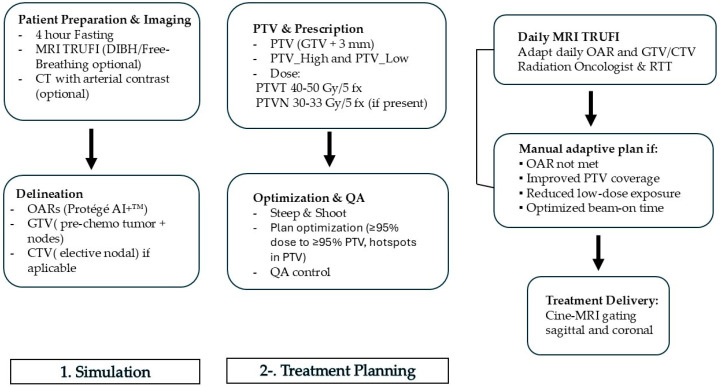
Schematic workflow of MR-guided radiotherapy using the MRIdian^®^ system. The process begins with patient preparation and imaging, including fasting, TRUFI MRI in deep inspiration breath hold (DIBH) or free-breathing, and optional contrast-enhanced CT. Organs at risk (OARs), gross tumor volume (GTV), and clinical target volume (CTV) were delineated, followed by planning target volume (PTV) generation and dose prescription. Treatment planning was performed with step-and-shoot IMRT and optimized to ensure ≥95% of the prescribed dose covered ≥95% of the PTV, allowing hotspots within PTV_High. Daily MRI recontouring of OARs and target volumes, and manual adaptive plans were generated if OAR constraints were exceeded, PTV coverage improved, low-dose exposure reduced, or beam-on time optimized. Treatment delivery was performed with cine-MRI gating in sagittal and coronal planes.

The clinical workflow was performed using the MRIdian A3i^®^ MR-guided radiotherapy system (ViewRay Inc., Oakwood, GA, USA) [18]. As shown in Figure 1, patients underwent arterial-contrast CT and TRUFI MRI simulation following a 4-h fasting period, using a Deep Inspiration Breath Hold (DIBH) protocol when feasible, or free-breathing if DIBH was not possible. Contrast-enhanced arterial-phase CT was performed at the treating physician’s discretion. Organs at risk (OARs) were delineated using Contour Protégé AI+™ (MIM Software^®^ v1.1.3) and reviewed by a radiation oncologist. Target volume delineation followed international consensus guidelines, including the NRG Oncology contouring atlas [19] and ESTRO-ACROP recommendations [20]. Table 1 shows organs at risk (OAR) for pancreas stereotactic MRI-guided radiotherapy in 5 fractions.

The gross tumor volume (GTV) encompassed the pre-chemotherapy tumor and involved lymph nodes. Elective nodal irradiation (ENI) was initially discretionary but later incorporated as a clinical target volume for nodal areas (CTVN) based on emerging evidence. When no CTVN was defined, a 3 mm isotropic margin was added to the GTV to create the planning target volume (PTVT). If a CTVN was present, a 3 mm margin was added to generate the corresponding PTV (PTVN), with an integrated boost applied to the GTV plus an additional 3 mm margin.

For each PTV, two sub-volumes were generated: a high-dose PTV (PTV_High, PTV optimized, or PTV_opt), defined as PTV − (OAR + 3 mm), and a low-dose PTV (PTV_Low), defined as (OAR + 3 mm) − PTV. Prescribed doses to the PTVT ranged from 40 to 50 Gy in five fractions over one week, while PTVN, if present, received 30 to 33 Gy in five fractions. Planning aimed to deliver ≥95% of the prescribed dose to ≥95% of the PTV, following inhomogeneities (hotspots) of 120–140% strictly confined to PTV_High.

Daily, OARs were recontoured by a radiation oncologist in collaboration with a radiation therapist (RTT). Step-and-shoot IMRT plans were adapted manually based on one or more of the following criteria: OAR dose constraints not met, improved PTV coverage relative to the reference plan, reduced low-dose exposure (≤40% of the prescribed dose), or optimization of beam-on time. Real-time gating was performed for each fraction using cine-MRI (bSSFP sequences) in the sagittal plane at 4 frames per second. Secondary quality assurance was conducted for each fraction using ArcCHECK^®^-MR.

#### Follow-Up and Outcome Assessment

Follow-up included contrast-enhanced CT or MRI at 6–8 weeks post-treatment, and subsequently every 3 months. PET-CT was recommended in cases of suspected in-field recurrence. Surgical reevaluation was performed based on imaging response and multidisciplinary tumor board review. Post-SMART chemotherapy was administered at the discretion of the medical oncologist.

Toxicities were prospectively recorded and graded using the Common Terminology Criteria for Adverse Events (CTCAE v5.0). Acute toxicity was defined as occurring within 3 months of treatment completion; late toxicity was defined as occurring thereafter.

Clinical outcomes were defined as follows:

- Local control (LC): Absence of progression in the primary tumor and regional lymph nodes (RECIST v1.1).
- Overall survival (OS): Time from diagnosis to death or last follow-up.
- Distant progression-free survival (DPFS): Time from diagnosis to first distant metastasis.
- Progression-free survival (PFS): Time from diagnosis to progression, death, or last contact.
- Treatment-free interval (TFI): Time from the last SMART fraction to the appearance of distant metastases.

### 2.3. Statistical Analysis

Demographic, clinical, and treatment-related data were prospectively collected. Categorical variables were summarized using absolute and relative frequencies with confidence intervals where appropriate. Survival outcomes were estimated using the Kaplan–Meier method. All statistical analyses were performed using SPSS^®^ 30.0.0 software.

## 3. Results

A total of 28 patients were included in the analysis. Baseline characteristics are summarized in Table 2. The median age was 67 years (range, 43–86), with 11 patients (39.3%) being women and 17 (60.7%) men. Seven patients (25%) were classified as borderline resectable, and 21 (75%) as locally advanced pancreatic cancer (LAPC). ECOG performance status was 0 in 15 patients (53.6%) and 1 in 13 patients (46.4%). 6 patients had biliary stent placement before treatment.

FOLFIRINOX was the most frequently administered chemotherapy regimen (57.1%), followed by gemcitabine/nab-paclitaxel (24.6%) and gemcitabine monotherapy. The median number of chemotherapy cycles was 6 (range, 4–12). Tumors were most commonly located in the pancreatic head (71.4%), with a median tumor size of 32.7 mm (range, 15–58 mm). CA 19-9 levels were available in 10 patients (35.8%), with a median value of 1029 U/mL (range, 136–1923). Nine patients (32.1%) had lymph node-positive disease. The median time from diagnosis to initiation of chemotherapy was 2.7 ± 2.3 months.

### 3.1. Treatment Characteristics

A total of 140 fractions were delivered, all with daily online adaptation using a 0.35T MR-guided linear accelerator. The median time from MRI simulation to treatment initiation was 11 days (range, 1–21), and the median overall treatment duration was 5.9 ± 2.0 days, with treatments performed on consecutive days (i.e., daily).

Dose prescriptions included 50 Gy in five fractions (50% of patients), 40 Gy (32.1%), and 45 Gy (17.9%). The median PTV V95% was 90.6% (range, 64.4–99%), and the median PTV Dmean was 48.7 Gy (range, 39.9–62.5 Gy). For the optimized PTV (PTV_opti), the median V95% was 94.0% (range, 58.7–99%), and the Dmean was 55.1 Gy (range, 44.6–65.6 Gy).

The breath-hold technique was used in 23 patients (81.1%), while 5 patients (18.9%), mostly elderly, were treated under free-breathing conditions. In these patients, the PTV margin was not increased, and remained at 3 mm.

All patients were immobilized using a footrest and extended arms position, without abdominal compression. The median treatment session time, defined as couch time, was 45 min.

### 3.2. Toxicity and Outcomes

Four patients (14.3%) experienced grade 2 acute abdominal pain. Two patients developed grade 2 cholangitis, attributed to pre-existing biliary stents. One patient presented with upper gastrointestinal bleeding three months after receiving 50 Gy, requiring transfusion due to stent displacement and tumor progression. No grade ≥3 toxicities were attributed to SMART. One patient reported grade 2 late abdominal pain.

### 3.3. Oncologic Outcomes

The median follow-up from diagnosis was 12.25 ± 4.97 months and 7.42 ± 3.51 months since SMART. Local recurrence occurred in three patients (10.7%), including one in-field failure confirmed by pathology in a patient with concurrent peritoneal carcinomatosis. Despite initial encouragement for surgery, no patient underwent resection during the study period, mainly due to persistent vascular involvement, medical contraindications, or patient preference.

**Figure 2:**
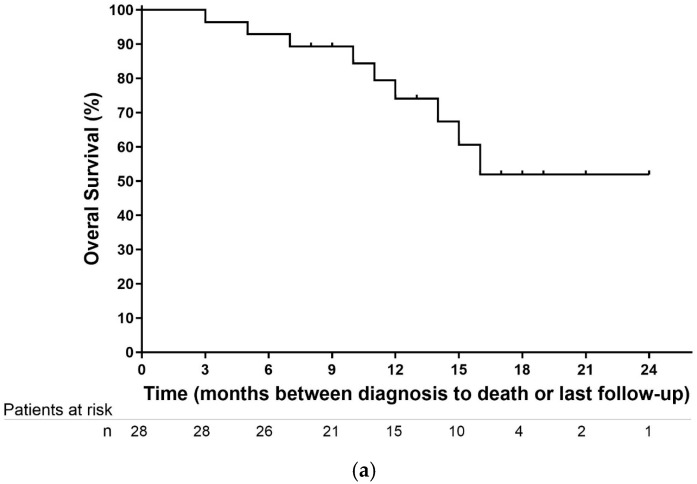

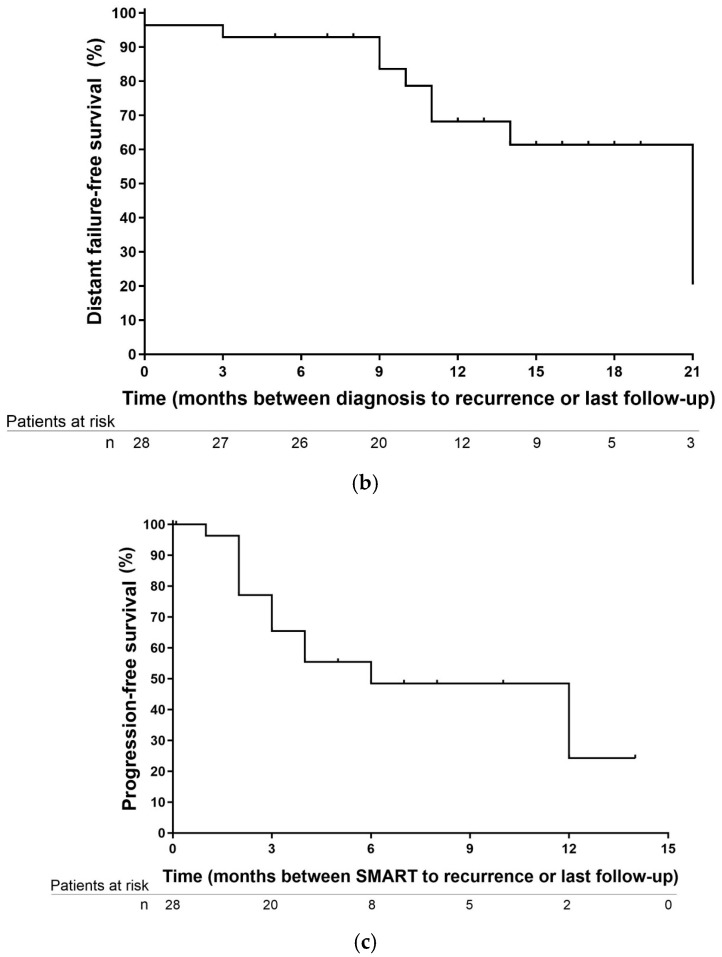
Kaplan–Meier plots. (**a**) Overall survival (OS): Time from diagnosis to death or last follow-up. (**b**) Distant progression-free survival (DPFS): Time from diagnosis to first distant metastasis. (**c**) Progression-free survival (PFS): Time from diagnosis to progression, death, or last contact.

Six-month local control (LC) was 82.1% from diagnosis and 89.3% from SMART. Median OS from diagnosis was not reached. OS at 6 and 12 months was 89.3% (95% CI, 78.6–90.1%) and 74.1% (95% CI, 55.7–74.1%), respectively (Figure 2a).

Median treatment-free interval (TFI) was 5.7 months (range, 1–12). Median distant progression-free survival (DPFS) from diagnosis was 21 months, with 6- and 12-month rates of 92.86% (95% CI, 84.63–100%) and 68.17% (95% CI, 71.77–88.87%), respectively (Figure 2b).

Median progression-free survival (PFS) was 11.5 ± 5.3 months; 6- and 12-month PFS rates were 89.3% (95% CI, 82.2–100%) and 65.6% (95% CI, 49.8–89.4%), respectively (Figure 2c).

Distant metastases were observed in 10 patients, with the peritoneum (60%) and liver (30%) as the most frequent sites. At the time of analysis, nine patients had died: 4 due to metastatic progression (44.4%), 2 from local progression (22.2%), 2 from non-oncological causes (22.2%), and 1 from unknown causes (11.1%).

## 4. Discussion

This prospective observational study reports the first clinical experience with stereotactic MR-guided adaptive radiotherapy (SMART) for pancreatic ductal adenocarcinoma (PDAC) in Spain. Our results demonstrate that SMART is feasible, safe, and potentially effective for borderline resectable and locally advanced disease. The 6-month local control rate of 89.3%, together with the absence of grade ≥3 toxicity, is consistent with international experiences and highlights the reproducibility of MR-guided workflows in daily clinical practice.

Delivering ablative doses (>10 Gy per fraction) has been shown to partially overcome the classical concept of radio resistance in PDAC, challenging the linear–quadratic model and the 4Rs of radiobiology. High-dose SBRT induces enhanced DNA damage, disrupts tumor vasculature, and prevents repopulation [21]. Clinical data indicate that PDAC patients receiving a biologically effective dose (BED) greater than 70 Gy compared to 50.4 Gy achieve superior overall survival (17.8 vs. 15.0 months, *p* = 0.03, respectably) [22]. In contrast, early phase I SBRT studies, such as Koong et al. [23] with single-fraction 25 Gy, reported excellent local control but unacceptable gastrointestinal toxicity, largely due to motion uncertainties and lack of adaptive planning.

The integration of MRI with linear accelerators represents a crucial advancement in overcoming these limitations. Compared with CBCT, MRI provides superior soft-tissue contrast, minimal motion artifacts, and no additional radiation exposure, while enabling deformable image registration, daily recontouring, online optimization, and real-time replanning [24]. In our cohort, daily adaptation was systematically implemented in all patients across 140 fractions, ensuring individualized optimization of target coverage and organ-at-risk sparing. This comprehensive adaptive workflow represents a major step forward compared with previously published series.

Target delineation in pancreatic SBRT is inherently challenging due to tumor and nodal variability. Following NRG Oncology [20] and ESTRO-ACROP [19] recommendations, we applied a dual-prescription strategy, delivering 40–50 Gy in five fractions to the gross tumor and 30–33 Gy to elective nodal areas. This approach balances microscopic disease coverage with gastrointestinal safety and may increase the probability of achieving R0 margins at common sites of involvement, particularly uncinate, neck, and vascular regions [25]. Importantly, only four patients experienced grade 2 gastrointestinal toxicity, mostly stent-related.

In terms of patient setup, immobilization and respiratory management strategies also impacted adaptation. In our elderly population, mild inspiration breath-hold was generally better tolerated and more reproducible than deep inspiration breath-hold. Although other groups [26,27,28,29,30,31] have reported that variations in breath-hold technique do not significantly affect adaptation outcomes, in our experience, changes in gastrointestinal anatomy remained the primary driver of plan modification.

Our data supports the extension of SMART use to borderline resectable and locally advanced pancreatic cancer in real-world populations. Since introducing SMART in Spain, new clinical opportunities have emerged, including oligometastatic disease, re-irradiation, and treatment of local recurrences. Retrospective series by Webiking et al. [26] demonstrated that ablative SMART (50 Gy) can achieve meaningful local control (68% at 1 year) and pain relief with minimal morbidity in oligometastatic settings. Similarly, Bryant et al. [27] and Chuong et al. [31] have reported encouraging local control rates (~88%) with SMART re-irradiation while maintaining acceptable toxicity profiles.

Although the median overall survival (OS) was not reached in our cohort, OS rates from diagnosis at 6 and 12 months were 89.3% (95% CI, 78.6–90.1%) and 74.1% (95% CI, 55.7–74.1%), respectively, which compare favorably with historical data such as the LAP07 trial [13], where the median OS with chemoradiotherapy was 15.2 months. Published experiences with stereotactic MR-guided adaptive radiotherapy (SMART) by Tringale et al. [17] and Bryant et al. [27] have reported comparable 1-year OS rates (80–100%), while long-term outcomes from the SMART trial [29] for borderline resectable (BRPC) and locally advanced pancreatic cancer (LAPC) showed 2-year OS rates of 53.6% from diagnosis and 40.5% from the start of SMART therapy, with minimal Grade ≥ 3 toxicity. These findings establish a growing body of prospective evidence supporting the efficacy and safety of SMART in this patient population.

Additionally, the observed treatment-free interval (TFI) of 5.07 months in our cohort may have important clinical implications. In an era increasingly focused on quality of life and minimizing treatment burden, the capacity of SMART to delay the need for systemic therapy—and thereby potentially reduce cumulative toxicity—is particularly relevant.

Nevertheless, limitations must be acknowledged. Our sample size was small, although appropriate for an initial clinical experience in Spain. The median follow-up of 7.4 months limits evaluation of long-term outcomes such as late toxicity and durable local control, though extended follow-up is ongoing. The absence of a comparator arm precludes definitive conclusions on the incremental benefit of MRI guidance over conventional SBRT or IMRT; randomized trials are needed. We did not report pathological response post-SMART due to the advanced age of our cohort and limited surgical experience, which is expected to improve with wider adoption of this technique. Finally, this experience reflects a single institution with substantial MRI-Linac expertise, which may limit generalizability.

## 5. Conclusions

In conclusion, our preliminary findings suggest that SMART for pancreatic adenocarcinoma is a feasible and safe technique that allows for dose escalation while minimizing toxicity. The strategic selection of prescription dose to maintain a BED > 70 Gy, balanced against duodenal dose constraints, appears to be a key factor in achieving promising local control with acceptable toxicity. However, given the limited follow-up, further research with longer observation periods is warranted to validate these results and to better define the role of SMART in the multidisciplinary management of pancreatic cancer.

## Figures and Tables

**Table 1:** Organ at Risk (OAR) for pancreas stereotactic MRI-guided radiotherapy in 5 fractions.

Organ at Risk (OAR)	Dose Constraint
Esophagus, Stomach, Duodenum,	V33 < 0.05 cc
Small bowel	V30 < 5 cc
Large Bowel	V52.3 < 0.035 cc
	V32.5 < 20 cc
Liver	Dmean < 15 Gy
Aorta	V47 < 10 cc
Spinal Cord	Dmax (0.035 cc) < 12 Gy
Kidneys	V17.5 < 200 cc

**Table 2:** Patient characteristics (n = 28).

Characteristic	Value
Age, median (range)	67 (43–86)
Sex	Male 17 (60.7%) Female 11 (39.3%)
Tumor location	Head 20 (71.4%) Body/Tail 8 (28.6%)
ECOG	0: 15 (53.6%) 1: 13 (46.4%)
Stage	Borderline: 7 (25%) LAPC: 21 (75%)
Clinical T	T1: 1 (3.6%) T2: 9 (32.1%) T3: 6 (21.4%) T4: 12 (42.9%)
Tumor size, median (mm)	32.7 (15–58)
Clinical N	0: 19 (67.9%) 1: 9 (32.1%)
CA 19-9, median (U/mL)	1029 (136–1923)
Chemotherapy regimen	FOLFIRINOX: 16 (57.1%) Gemcitabine/Abraxane: 7 (24.6%) FOLFIRI: 1 (3.6%) Other: 4 (14.3%)
Chemotherapy cycles, median	6 (4–12)
Radiation dose	40 Gy: 9 (32.1%) 45 Gy: 5 (17.9%) 50 Gy: 14 (50%)
Motion management	DIBH: 23 (81.1%) Free breathing: 5 (18.9%)

## Data Availability

The raw data supporting the conclusions of this article will be made available by the authors on request.

## Abbreviations

SMART	Ablative Stereotactic MR-guided Adaptive Radiation Therapy
BED	Biologically Effective Dose
BRPC	Borderline Resectable Pancreatic Cancer
bSSFP	Balanced Steady-State Free Precession
CA 19-9	Carbohydrate Antigen 19-9
CT	Computed Tomography
CTCAE	Common Terminology Criteria for Adverse Events
CTV	Clinical Target Volume
DFFS	Distant Failure-Free Survival
DIBH	Deep Inspiration Breath Hold
DVH	Dose–Volume Histogram
ECOG	Eastern Cooperative Oncology Group
ENI	Elective Nodal Irradiation
GTV	Gross Tumor Volume
IMRT	Intensity-Modulated Radiation Therapy
LAPC	Locally Advanced Pancreatic Cancer
LC	Local Control
MRI	Magnetic Resonance Imaging
MRgRT	Magnetic Resonance-guided Radiotherapy
MR-LINAC	Magnetic Resonance Linear Accelerator
OAR	Organ at Risk
OS	Overall Survival
PDAC	Pancreatic Ductal Adenocarcinoma
PET-CT	Positron Emission Tomography–Computed Tomography
PFS	Progression-Free Survival
PTV	Planning Target Volume
PTV_High/PTV\_opt	High-dose Planning Target Volume (Optimized)
PTV_Low	Low-dose Planning Target Volume
RTT	Radiation Therapist
SBRT	Stereotactic Body Radiation Therapy
TFI	Treatment-Free Interval
TRUFI	True Fast Imaging with Steady-State Precession
ULN	Upper Limit of Normal
